# Non-Consumptive Predator Effects Shape Honey Bee Foraging and Recruitment Dancing

**DOI:** 10.1371/journal.pone.0087459

**Published:** 2014-01-27

**Authors:** Allison Bray, James Nieh

**Affiliations:** Division of Biological Sciences, Section of Ecology, Behavior, and Evolution, University of California San Diego, La Jolla, California, United States of America; University of Arizona, United States of America

## Abstract

Predators can reduce bee pollination and plant fitness through successful predation and non-consumptive effects. In honey bees, evidence of predation or a direct attack can decrease recruitment dancing and thereby magnify the effects of individual predation attempts at a colony level. However, actual predation attempts and successes are relatively rare. It was not known if a far more common event, just detection of a predator, could inhibit recruitment. We began by testing honey bees' avoidance of the praying mantis (*Tenodera sinensis*). Larger predators (later mantis instars, ≥4.5 cm in body length) elicited significantly more avoidance (1.3 fold) than smaller mantis instars. Larger instars also attempted to capture honey bees significantly more often than did smaller instars. Foragers could detect and avoid mantises based upon mantis odor (74% of bees avoided an odor extract) or visual appearance (67% avoided a mantis model). Finally, foragers decreased recruitment dancing by 1.8 fold for a food source with a live adult mantis, even when they were not attacked. This reduction in recruitment dancing, elicited by predator presence alone, expands our understanding of predator non-consumptive effects and of cascading ecosystem effects for plants served by an important generalist pollinator.

## Introduction

Predators do not need to kill to exert significant effects on prey because non-consumptive effects of predation amplify predator effects beyond actual kill rates [1], [2]. Thus, it is important to know precisely what triggers these non-consumptive effects, if these triggers are rare or common, and how the resulting effects can be amplified. By altering prey behavior, non-consumptive effects can have broad, cascading ecosystem consequences [3] that shape food webs and affect primary production [1], [4]. In the case of pollination, a key ecosystem service [5], non-consumptive effects can alter plant-pollinator mutualisms and therefore have widespread influences [6]. For example, bees avoid flowers upon which they have experienced an unsuccessful predation attempt [7], [8]. Bees also avoid other indicators of predation risk: dead bees [7], [9], bee hemolymph [10], dead crab spiders [7], [11], and living crab spiders [12], [13]. Such avoidance can decrease plant fitness. Predator presence led to decreased pollinator visitation and diminished seed output in *Leucanthemum vulgare* [14] and reduced seed set and fruit mass in *Rubus rosifolius* [15].

Non-consumptive predation effects can also shape the emergent behavior of mass recruiting pollinators. Honey bees perform recruitment dances to recruit nestmates to food resources [16]. This recruitment can enhance colony fitness [17], [18] and should contribute to plant fitness by increasing pollination for rich food patches. However, non-consumptive effects can decrease honey bee recruitment. Abbott and Dukas [19] showed that honey bees (*Apis mellifera*) exposed to a recently killed bee on a feeder would dance less than those returning from control feeders. Bees that were attacked at a feeder by conspecifics also reduce recruitment dancing [20]. Similarly, *A. florea* workers exhibited alarm behavior and reduced waggle dancing upon the approach of a potential predator, a human [21]. Recently, Tan et al. [22] showed that individual foragers of an Asian honey bee, *A. cerana*, continue to visit a moderately dangerous feeder with a hornet predator. However, the colonies of these foragers allocate significantly fewer foragers to such a dangerous feeder. The researchers hypothesized that individual bees continue to forage, but dance less after encountering the predator.

It is not known if detecting a predator, rather than concrete evidence of predation or being attacked, can inhibit waggle dancing. This is a key gap in our understanding because encounters with predators are far more common than successful predation [13]. Crab spiders attack 4–11% of visits by bumble bees and honey bees to milkweed umbels and successfully capture bees in only 0.4–1.7% of cases [12], [23]. Natural rates of predator detection are much higher than these attack rates. Overall, the presence of live predators on flowers reduces the visits of insect pollinators by 36% [24]. In our data, 67% of honey bees avoided a feeder with a live predator that did not attack (see below). The effect of predators on colony foraging activation and pollination should thus be greater than previously suspected if honey bees can reduce recruitment when they detect predators, not only when they are directly attacked or sense evidence of predation.

It is also unclear how honey bees detect predators, which are often cryptic. There are two main modalities, vision and olfaction. Goncalves-Souza et al. [15] used a model crab spider and elicited general avoidance by a guild of pollinating insects, though the effect on honey bees is not clear because *A. mellifera* visited at a relatively low rate. Romero [24] conducted a meta-analysis and found that artificial spider models decreased pollinator visitation more strongly than live crab spiders. This effect is significant for Hymenoptera as a whole, but may not hold for *A. mellifera* considered separately.

Odor is also important for predator detection [25]. Significantly more stingless bees approached flowers when all odor (floral and spider odor) was excluded [26]. However, excluding both flower and spider odor did not alter honey bee attraction [27]. Reader et al. [28] suggested that honey bees could avoid spider odor because bees avoided flowers that a spider had walked upon and may have deposited spider odor. A test of whether honey bees will avoid an extract of predator odor would directly answer this question.

**Figure 1:**
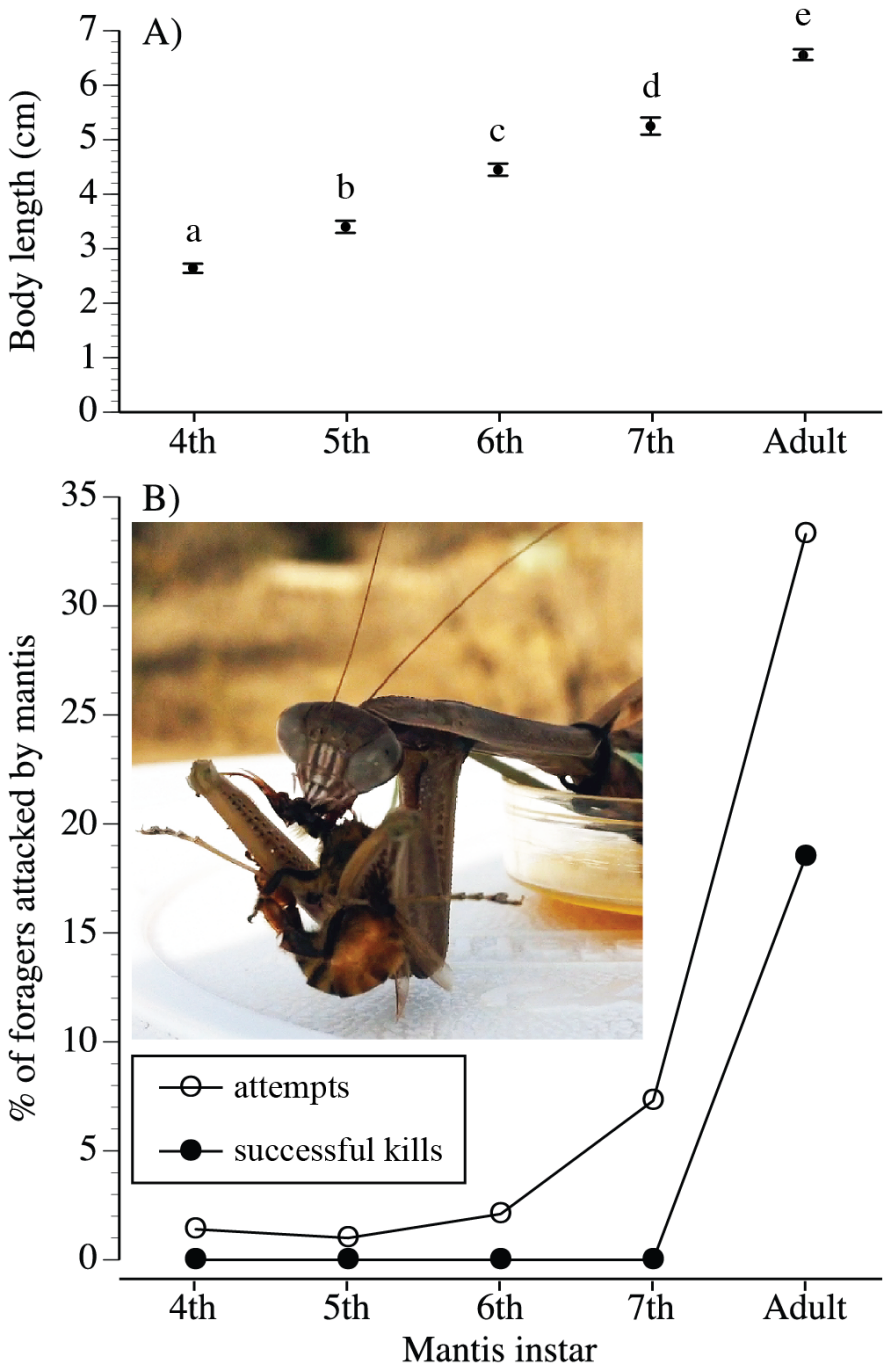
Effect of mantis developmental stage (size) on predation attempts and successes. The 7^th^ instar is the final instar before adulthood. A) Sizes of different instars (different letters indicate significant differences), standard error bars shown. B) Percentage of foragers attacked (open circles) and successfully killed (filled circles) by mantises. The inset image (online version in color) shows a tethered mantis eating a captured bee on the feeder. Mantises could place themselves directly on the feeder, as shown, but also positioned themselves outside the feeder, within an 8 cm radius.

Finally, larger predators can be more dangerous [29], and thus predator size should play a role in the magnitude of prey avoidance. For example, bigger predators elicit significantly greater avoidance in prey fish and iguanas [30] and in birds [29], [31]. To date, no studies have tested the effect of predator size on avoidance behavior by insect pollinators. Ideally, such a study would use different sizes of the same predator species. Praying mantids are widespread generalist predators that prey upon honey bees, bumble bees, and wasps [32]. Mantises also markedly increase in size throughout their life cycle (Fig. 1A), allowing us to determine how increasing size (later instars) in the same predator species affects prey avoidance.

The mantis, *Tenodera sinensis*, was introduced to the United States from eastern Asia in 1896, and now occurs throughout temperate North America [33]. This species preys upon honey bees visiting flowers [34] and reduces hymenopteran density [35], perhaps through successful predation, non-consumptive effects, or both. We began by testing if *T. sinensis* would elicit bee avoidance. We first tested the hypothesis that larger predators (successive mantis instars) elicit a stronger avoidance response. We then determined the relevant sensory modalities: if bees could use mantis visual or olfactory cues alone to detect and avoid these predators. Finally, we tested if honey bees would reduce recruitment dancing in response to detecting a predator, not just to the less common situations of being directly attacked or detecting evidence of predation.

## Materials and Methods

### General methods

We used a total of 16 colonies of European honey bees, *A. mellifera ligustica*, at the UCSD Biological Field Station (La Jolla, California, USA) between July 2011 and August 2013, from 12:00 to 15:00 on experimental days. We conducted feeder choice experiments throughout the year and dance experiments during times of relative food dearth (fall and early winter) when bees are more likely to recruit for feeders. The full colonies (*n* = 14) were housed in standard 10-frame Langstroth hives. Observation colonies (*n* = 2) each contained three combs [design of 16] and were placed inside a trailer with a 3 cm inner diameter tube (20 cm long) to allow bees to enter and exit from outside. We trained bees by presenting approximately 5 ml of unscented 2.5 M sucrose solution (65% sucrose w/w) in a 4 cm diameter yellow plastic dish. We used this very rich food to provide a reward that would consistently elicit interest among foragers, even when natural food sources were abundant. We placed the feeder at the center of a foraging platform, a 20 cm diameter white plastic disk, atop a 1 m high tripod near the nest entrance and progressively moved it away once bees began to feed (methods of [16]). Wedid not use any training odors and thoroughly cleaned all equipment with Alconox laboratory detergent after each trial to remove potential odors. We used standard methods to rear mantises (*T. sinensis*) from egg cases, feeding mantises a diet of fruit flies and crickets [36].

### Feeder choice tests

**Figure 2:**
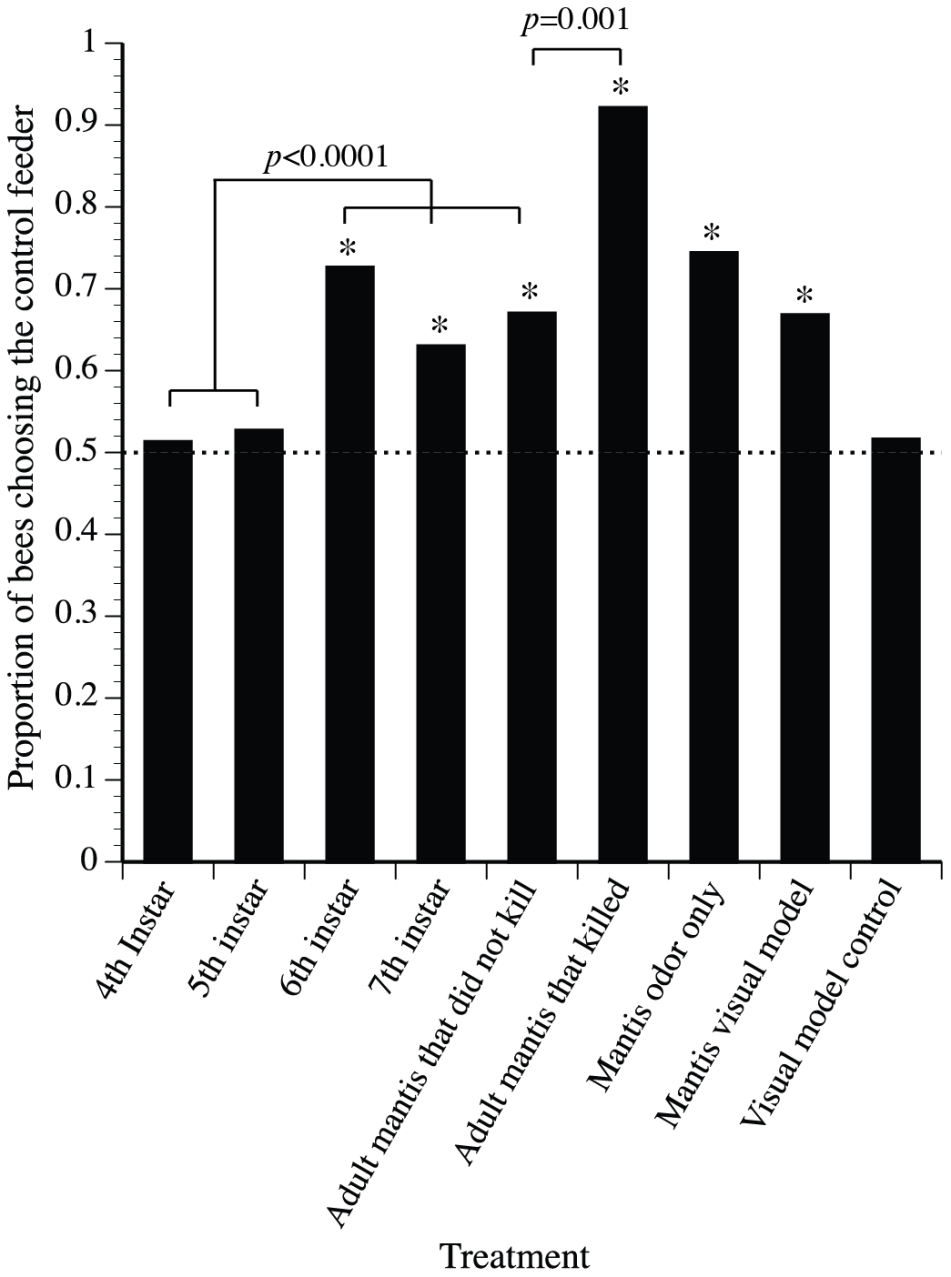
Effects of mantis developmental stage, appearance, and odor on bee foraging choices. Only adults successfully killed bee foragers, and we therefore divide adults into the two groups shown. The dashed line shows the null expectation, equal visitation to both feeders. The only two significant contrasts are shown: earlier vs. later mantis instars and successful vs. adults that did not kill a bee (marked with lines and *p*-values). Asterisks indicate significant avoidance of the dangerous feeder (binomial probabilities, p≤0.001, see Results).

We worked with 14 full bee colonies and used one colony at a time. For each trial, we trained 15 marked bees to a feeder placed approximately 10 m away from the focal colony. Once the bees were trained (approximately 5 visits/bee), we did not replenish the sucrose solution and allowed the feeder to become empty. We then removed the training feeder and set out two identical clean feeders, each containing approximately 1 ml of 2.5 M unscented sucrose, on separate, clean foraging platforms spaced 40 cm apart and equidistant from the focal nest. At the experimental feeder, we used an 8 cm long embroidery-thread tether tied around the thorax of a live mantis and attached at the other end with a 1 cm square of clear tape to the center of a white feeding platform, over which we centered the feeder (Fig. 1B). This tethering placed mantises within striking distance of foragers. The control feeder was identical but did not contain a mantis. We recorded bee choices over a 15 min trial and swapped feeder positions each 5 min to avoid potential site bias. We alternated the side (left or right) at which we placed the mantis at the beginning of each trial. Thus, over all trials, the mantis and control feeders were tested for equal amounts of time at both positions. To ensure the independence of choices and to eliminate potential social facilitation, we used plastic vials to immediately capture all bees that landed on either feeder and only counted choices made in the absence of other bees on or near the feeders. This capture technique did not release alarm pheromone because significantly more bees preferred the safe feeder where we made the most captures. Honey bees avoid their alarm pheromone at a feeder [10]. If capturing bees released alarm pheromone, foragers would have avoided the safe feeder. However, they preferred the safe feeder for all treatments except small mantises (4^th^–5^th^ instars) that were harmless (they made the fewest attempts to capture bees and never killed bees, Fig. 1) and the visual model control (a brown cylinder, Fig. 2). We recorded each bee's choice only once. We painted all captured bees on their thoraces with enamel paint before releasing them at the end of the trial and did not count the choices of any painted bees.

We used mantises of different instars (instars 4 to 7, and adult mantises) to test the effect of predator size on bee avoidance behavior. We measured mantis size based upon the largest dimension visible to bees: the length from the tip of the head to the end of the abdomen (instars≤7^th^) or to the end of the wings for adults (the 8^th^ instar) because adult wings extend slightly past the abdomen. We recorded attempts by mantises to catch bees, i.e. instances where a mantis touched a bee with its two raptorial forelegs, as well as successful bee kills. Only adults successfully killed bees. Because a kill can release bee alarm pheromone and the odors of bee body fluids, we separated the adult mantises into two categories: mantises that killed a bee and mantises that did not. Each mantis was used for an average of 5 trials, with an interval of several days between reuse. A mantis that killed a bee was never re-used to avoid the residual odors of a killed bee from potentially influencing other trials.

### Predator recognition cues

We tested bee avoidance of (1) an adult mantis (see above), (2) mantis odor, (3) mantis appearance (plastic model with no mantis odor), and (4) a mantis model control using the choice array described above. Hexane is an effective solvent for extracting insect cuticular hydrocarbon odors [37] and can be used to remove and subsequently restore insect olfactory recognition signals [38]. In harvester ants, solvent extracts of worker cuticular hydrocarbons elicit olfactory recognition from nestmates [39]. Behavioral assays show that hexane successfully extracts signal odors from exocrine glands of stingless bees [40], bumble bees [41], and honey bees [20]. To obtain mantis odor, we therefore added 3 frozen dead 4^th^ instar mantises and 10 4^th^ instar mantis exuviae to 10 ml of hexane in a clean glass bottle, agitated this at 400 rpm for 3 hours at 21°C, and separated out the hexane out into a new bottle stored at 4°C until use. The dead mantises and mantis exuviae were from mantises that were never exposed to honey bees and thus had no honey bee odor. During trials, we pipetted out 500 µl of the mantis odor extract (corresponding to the extract from half a mantis) onto a 2.5 cm diameter circle of Whatman #2 filter paper placed under the experimental feeder. The control was an equal volume of pure hexane on an identical clean filter paper placed under the control feeder. The mantis model (Safari Ltd. USA, Miami Gardens, Florida, 22169, USA) had the appearance of an adult mantis, and was 6.7 cm long, consistent with an adult *T. sinensis*. The model control was a brown 6.7 cm long and 1.9 cm high cylinder of the same approximate size as the mantis model [after design of 7].

### Effect on recruitment communication

To test the hypothesis that foragers would decrease dancing for a rich food source with a live predator, we alternately used two observation colonies and trained bees from the focal colony to an *ad libitum*, 2.5 M unscented sucrose feeder placed 1 m from the nest entrance. This short distance to the feeder reduced the cost of food collection for the bees, made the feeder a highly desirable food source, and encouraged recruitment dances. For such short distances, recruiting bees perform what is classically called the “round dance”[16], though recent studies suggest this is part of a continuum of behaviors to which the term “waggle dance” should be applied [42]. We counted the number of dance circuits, which are positively correlated with recruitment for both round and waggle dances and are the natural unit of dance information [16].

We conducted one trial per day, beginning with the “before” phase and ending with the “after” phase. For a trial, we first trained several bees to the feeder, painting each bee with a unique combination of color marks on its thorax so that it could be easily located and identified in the observation hive. In the “before” phase, we used a timer to record the amount of time that each bee waited to unload her collected food (unloading wait time) and counted the number of dance circuits performed by each bee. For the “after” phase, we tethered an adult mantis (as above) to the center of the before-phase feeding platform and used the before-phase feeder. Thus, the only change was adding the mantis. We then recorded the same data for each bee. In the control trials, each bee was also recorded dancing twice, but we did not add a predator in the “after” phase. Bees perform the same number of waggle circuits on each return to the nest for a good, unchanging food source [43]. We therefore expected bees to produce the same number of dance circuits for the control treatment and fewer dance circuits for the predator treatments: (1) mantis that killed a bee while other foragers were visiting the feeder and (2) mantis that did not attempt to attack a bee. In this experiment, we allowed multiple bees to simultaneously visit the feeder, and all mantis predation attempts were therefore successful because there were several potential victims for each attack. We never reused a successful mantis, which could carry odors of bee predation, in a later mantis trial.

### Statistics

We used a one-way ANOVA to test the effect of mantis instar on mantis length, and a Tukey-Kramer HSD test to determine which instars significantly differed from one another in length. For the foraging preference experiments, we used 2-tailed binomial tests (H_o_: *p* = 0.5). We used a Generalized Linear Model (GLM) with contrasts [44] to determine if there was an effect of mantis size (4^th^ and 5^th^ vs. 6^th^ and older instars), an effect of a mantis that killed a bee, and an effect of different cues (adult mantis vs. mantis visual cues, adult mantis vs. mantis odor, and mantis visual cues vs. mantis odors). Because of these multiple tests, we apply the Sequential Bonferroni procedure, indicating tests that pass as SB* [45]. For the dancing experiments, we used repeated-measures ANOVAs to test the effect of experimental phase and unloading wait time on the number of dance circuits. We used ANOVA to test for an effect of time between phases on changes in waggle dancing per individual. These data met parametric assumptions as determined through residual analyses. All statistical analyses were conducted with JMP v10 software.

## Results

### Effect of predator size

Mantis size influenced mantis hunting behavior and success. Mantises become significantly larger (greater length) in each subsequent instar (ANOVA, *F*_4,17_ = 250.76, *p*<0.0001), and all instars are significantly different from each other (Tukey HSD, *p*<0.05, Fig. 1A). Later mantis instars made significantly more predation attempts than earlier instars (GLM, Exponential distribution, Reciprocal link, Maximum Likelihood, *χ* ^2^_1_ = 8.36, *p* = 0.004). Only adult mantises successfully captured bee foragers (Fig. 1B).

Bees increasingly preferred the safe feeder as the mantis threat increased (Fig. 2). Each bee approached the feeder array and generally flew around both feeders, often within a few cm of the feeder before making a choice. There is a significant effect of mantis size on the proportion of bees choosing the control vs. the experimental feeder (GLM, binomial distribution, logit link, maximum likelihood, *χ* ^2^_7_ = 63.7, *p*<0.0001^SB*^). Bees avoided the larger mantises (instars 6–7 and adults), on average, 1.3 fold more than the smaller mantises (instars 4–5, GLM contrast *χ* ^2^_1_ = 22.32, *p*<0.0001^SB*^). Specifically, bees avoided the 6^th^ instar (*n* = 172, binomial *p*<0.0001^SB*^), 7th instar (*n* = 149, binomial *p* = 0.002^SB*^), and adults (*n* = 161, binomial *p*<0.0001^SB*^). Bees did not avoid the 4^th^ or 5^th^ instars (*n* = 296, binomial *p* = 0.68 and *n* = 252, binomial *p* = 0.41, respectively).

### Predator recognition cues

Bees significantly avoided the mantis visual model (Fig. 2, *n* = 262, binomial *p*<0.0001^SB*^) and mantis odor (*n* = 121, binomial *p*<0.0001^SB*^). As expected, bees did not avoid the mantis model control (*n* = 95, binomial *p* = 0.42). Bees equally avoided live adults, mantis visual appearance, and mantis odor. The GLM contrast tests for adult vs. model (*χ* ^2^_1_ = 0.09, *p* = 0.76), model vs. odor (*χ* ^2^_1_ = 2.21, p = 0.13), and adult vs. odor (*χ* ^2^_1_ = 2.68, p = 0.101) are all non-significant. Successful predation during a trial elicited a very strong avoidance response in subsequent foragers (Fig. 2, only 8% chose the mantis feeder, *n* = 51, binomial *p*<0.001^SB*^). Significantly more bees avoided an adult mantis that killed a bee than an adult mantises that did not kill a bee (GLM contrast *χ* ^2^_1_ = 18.29, *p*<0.001^SB*^).

### Effect on recruitment communication

Mantis presence reduced bee recruitment dancing. In this experiment, we trained bees to a very rich food source at a time of relative food dearth and then gave them no choice between safe and dangerous feeders. Thus, approximately 70% of trained bees continued to visit the feeder after we added a live adult mantis. The no-mantis control group produced the same number of dance circuits in the before and after phases (*F*_1,14_ = 2.16, *p* = 0.16). However, foragers exposed to the mantis that did not kill a bee significantly reduced dancing by 1.8 fold (*F*_1,36_ = 7.25, *p* = 0.01). Similarly, foragers exposed to the mantis that killed a bee significantly reduced dancing by 2.7 fold (*F*_1,14_ = 9.39, *p* = 0.008). There is no significant effect of unloading wait time on the number of dance circuits produced (*F*_1,141_ = 1.47, *p* = 0.23). On average, 42 min elapsed between before and after phases, and there is no significant effect of time delay between phases on changes in waggle dancing (*F*_1,70_ = 1.72, *p* = 0.19).

## Discussion

Our results demonstrate that predator detection alone is sufficient to inhibit honey bee recruitment dancing. Thus, the role of non-consumptive effects of predators in shaping colony foraging activation may be stronger than previously thought. Relatively rare events, evidence of successful predation or direct experience of attack, are not necessary to inhibit colony recruitment. Honey bees significantly reduced recruitment dancing by 1.8 fold in response to the presence of a live adult mantis that did not attack a bee. Exposure to evidence of bee predation reduced dancing by 2.7 fold. This is the first demonstration that predator presence alone can reduce recruitment dancing and that a live predator in the act of consuming a bee will also reduce recruitment dancing. The latter result is not surprising, but the former result expands our understanding of how predator presence affects colony foraging. In addition, it was not known if predator size matters to insect pollinators. We show that bees avoided larger mantis instars more than smaller ones. Such avoidance makes sense because larger mantis instars made significantly more attempts to capture bees and only adults, the largest instar, successfully captured bees. Finally, previous studies suggested that honey bees could avoid predator odor. Our experiments show that bees can detect and avoid a predator, a praying mantis, from its visual appearance alone or odor alone.

### Effect of predator size

Surprisingly, few studies have examined the role of predator size in eliciting avoidance by prey that are pollinators. Previous studies have found an effect of pollinator prey size on predator avoidance [24], [46], [47], although not in all circumstances [8]. We found that predator size also matters. Honey bee foragers will avoid mantises above a certain size (≥4.5 cm in body length, corresponding to instars 6 and older, Fig. 2). The ability of a mantis to catch certain prey depends upon mantis size: because mantises do not use venom, they must physically overpower their prey [48]. Smaller *T. sinensis* mantises rarely attempted to capture honey bees (Fig. 1B), and so bees should discount them as a threat, as shown in our avoidance experiment (Fig. 2). We use the term “size” as a trait that naturally encompasses other traits, such as visual size, level of olfactory cues, and strength. Bees may use multiple information sources to discriminate size. For example, they may use mantis visual size and odor level if larger mantises produce more mantis odor.

In addition, bees might have responded to the results of attack behavior that typify the different mantis sizes (Fig. 1B). Bees could have avoided the smell of bee alarm pheromone, if this was released during mantis predation attempts (defined as a mantis touching but not killing a bee). The effect of this potential alarm pheromone release on our results is likely weak because, out of 1021 bees tested, only 0.7%, 0.5%, 0.6% 2.7%, and 4.8% of bees respectively experienced predation attempts from 4^th^, 5^th^, 6^th^, and 7^th^ instars and adult mantises. Moreover, the 4^th^, 5^th^, and 6^th^ instars attacked bees at approximately the same rate, but only the 6^th^ instar elicited significant bee avoidance (Fig. 2).

Honey bees were capable of visually detecting even the smallest mantis instar. Honey bees have excellent color vision [49], and the instars were a brown color that contrasted against the white background of the feeder platform. In addition, bees have sufficient visual resolution to spatially discriminate the mantis instars. Each bee generally flew around and came within a few cm of each feeder before making a choice. Honey bees require a minimum visual edge length of 3° for spatial discrimination [50]. At a distance of 20 cm away (equidistant between the two feeders in the choice experiment) the mantises presented average visual angles of 7°, 10°, 13°, 15°, and 18°, based upon the lengths of the 4^th^ instar-adult mantises. Bees should be able to discriminate the smallest instar even at a distance of 50 cm away (corresponding to a 3° edge length). It is unclear if bees used olfaction to detect the different mantis instars. However, vision alone is sufficient for a bee to detect and avoid a plastic mantis model with no mantis odor (Fig. 2). Thus, a failure to detect the smaller mantis instars is unlikely to account for our results.

### Predator recognition cues

Bees likely use olfaction and vision when trying to detect a predator. Studies have demonstrated that bees can avoid dead [7], [11] and living crab spiders [12], [13], which provide both olfactory and visual cues. Olfaction allows native Australian bees (but not honey bees) to avoid flowers with the crab spider, *Thomisus spectabilis* [26]. Reader et al. [28] suggested that honey bees may avoid spider odor: bees avoided flowers upon which a spider had walked and could have deposited spider odor and spider silk.

Some bee species can detect predators using vision alone. Bumble bees can learn to avoid the visual appearance of artificial crab spiders [51]. Romero [24] conducted a meta-analysis and found that artificial spider models decreased pollinator visitation more strongly than live crab spiders. This effect holds for Hymenoptera as a whole, but it is not clear if it is significant for *A. mellifera* considered separately. Goncalves-Souza et al. [15] simulated a crab spider with a model and elicited general avoidance by a guild of pollinating insects. However, it was not clear if the model predator led to honey bee avoidance because *A. mellifera* visited at a relatively low rate: 79-fold fewer visits than stingless bees, the most abundant hymenopteran visitors. It should be noted that some studies do not support visual predator detection. This may depend upon the realism of the model used. Pollinators, including bees, did not avoid an artificial paper spider placed on inflorescences [11].

In our study, honey bees avoided the mantis model (visual but no olfactory cues) and the mantis hexane extract (olfactory but no visual cues). Aversion was not significantly different between these cues or exposure to a living adult mantis (Fig. 2). Thus, visual or olfactory detection alone is sufficient to trigger avoidance, a useful strategy when dealing with cryptic ambush predators. Our mantis model may not have simulated the complete visual appearance of a mantis to a bee, but it was sufficient to elicit bee avoidance. Such avoidance is likely due to an assessment of risk, rather than simply neophobia, the avoidance of unfamiliar objects [52] because honey bees did not avoid the model control (Fig. 2). This agrees with earlier results showing that honey bees would avoid a dead spider but not a control plastic cylinder placed on a feeder [7]. Thus, our mantis model was sufficient to elicit a strong avoidance response in honey bee foragers (Fig. 2), in agreement with other studies, which show insect pollinators avoiding artificial spiders [15], [24].

Despite the risk, on average, 31% of foragers visited the feeder with an adult mantis, mantis odor, or mantis model (Fig. 2), perhaps because of the excellent reward provided (2.5 M sucrose solution). Animals are more likely to forage in high risk patches when the risky patches provide a good reward [6], [53] and bees have been shown to take more risks when colony energy supplies are low [54]. But sometimes the risk is too great. Only 8% of foragers visited the feeder with a mantis that killed a bee. This stronger avoidance may be due to the release of bee alarm pheromone or internal fluids as the mantis tore the bee apart (Fig. 1B). Honey bees will avoid the odor of honey bee hemolymph and alarm pheromone at a food source [10]. It may seem counterintuitive for bees to avoid cues that could signal a satiated and therefore unthreatening predator. However, a single bee kill does not guarantee predator satiation. We observed instances where a praying mantis would eat two or three bees within minutes of each other.

### Effect on recruitment communication

**Figure 3:**
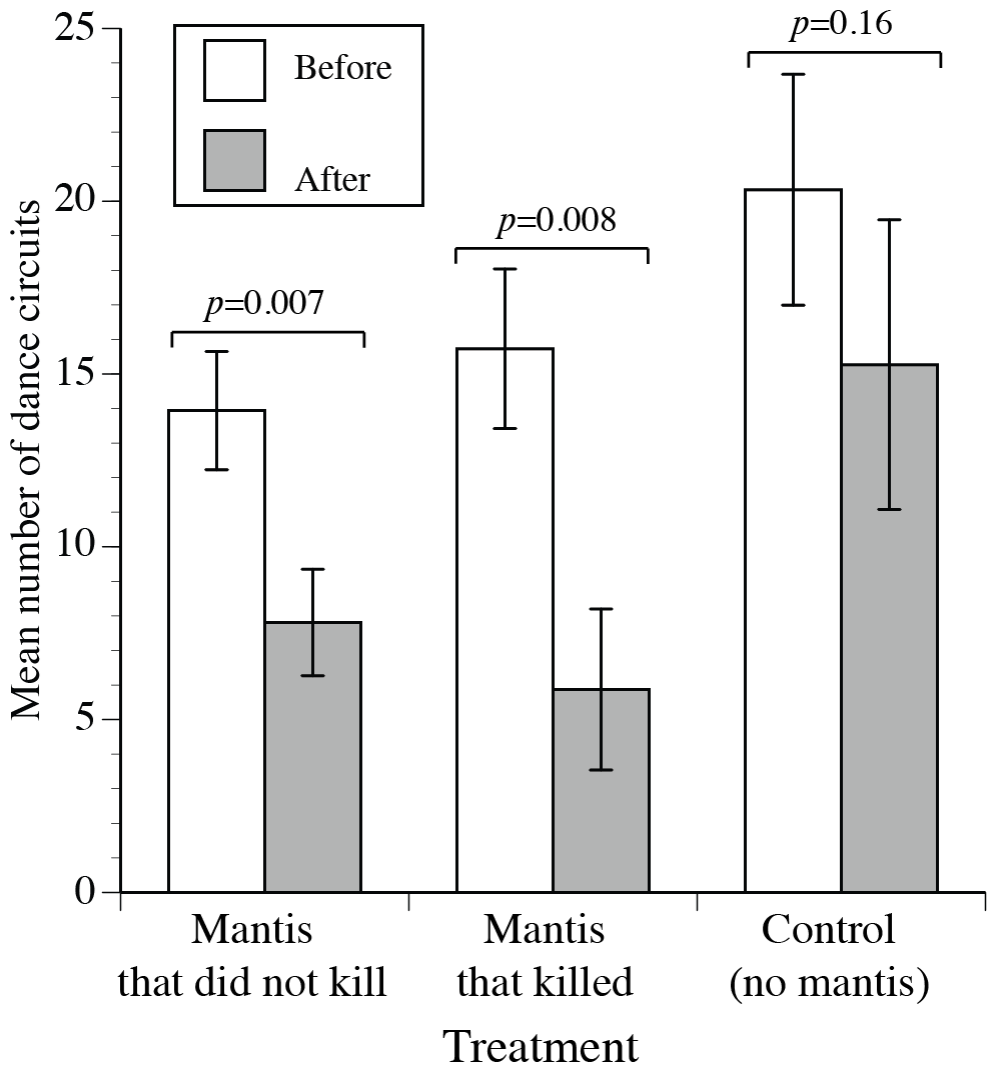
Effects of encountering mantis predators at a rich food source on honey bee recruitment dancing. The mean number of dance circuits per forager nest visit is shown *before* (white bars) and *after* the treatment (gray bars). Standard error bars are shown. The *p*-values for comparisons between phases are shown above each treatment.

Finally, colony foraging is a collective response that results from individual foraging choices and the ability of foragers to recruit nestmates. Recruitment dancing is tuned to food quality [43] and can be altered by a neuromodulator, octopamine, that may change this perception of reward quality [55]. We propose that predation risk can also modulate individual dancing. For example, direct experience of attack at a food source can reduce subsequent waggle dancing [20] and potential evidence of predation, such as a dead bee on a food source can reduce waggle dancing [19]. Here, we demonstrate that mere presence of a predator alone is sufficient to reduce recruitment dancing (Fig. 3). Honey bees therefore alter their foraging and recruitment behavior according to risk, not simply in response to predator attacks, but also to the possible threat of attack.

This finding that honey bees can reduce recruitment dancing solely in response to detecting a predator expands our understanding of how non-consumptive predation effects can shape colony foraging. Bees generally experience low natural attack rates (4–11% of visits) and relatively few are successfully captured [0.4]–[1.7% of visitors, 12,23]. However, this does not mean that predators have a minor effect on bee pollination. Through non-consumptive effects, live predators reduced overall insect pollinator visitation, including many bee species, by 36% [24]. After encountering live hornet predators at food, colonies of an Asian honey bee, *A. cerana*, decreased foraging at a low danger food patch. In contrast, individual foragers did not decrease visits to the low danger patch [22]. The greater avoidance shown by *A. cerana* colonies could arise if individuals continue to forage at dangerous patches but dance less and therefore recruit fewer nestmates. We show that this dance reduction occurs in individual *A. mellifera* foragers.

The effect of predator detection on colony-wide recruitment dancing and recruitment remains largely unknown. Studying this larger phenomenon is important because avoidance of predators could affect how colonies use their foraging landscape, potentially leading to changes in pollination patterns, and decreasing plant reproductive fitness. For plants, these effects upon the plant-pollinator mutualism may be complex because predators who repel pollinators may also decrease herbivore damage [56], [57]. However, bee colonies should generally benefit from being able to more carefully allocate their workforce.
